# Gene Cluster Profile Vectors: a method to infer functionally related gene sets by grouping proximity-based gene clusters

**DOI:** 10.1186/1471-2164-12-S2-S2

**Published:** 2011-07-27

**Authors:** Vikas Rao Pejaver, Sun Kim

**Affiliations:** 1 School of Informatics and Computing, Indiana University, 150 S Woodlawn Ave, Bloomington, IN 47405, USA

## Abstract

**Background:**

Proximity-based methods and co-evolution-based phylogenetic profiles methods have been successfully used for the identification of functionally related genes. Proximity-based methods are effective for physically clustered genes while the phylogenetic profiles method is effective for co-occurring gene sets. However, both methods predict many false positives and false negatives. In this paper, we propose the Gene Cluster Profile Vector (GCPV) method, which combines these two methods by using phylogenetic profiles of whole gene clusters. The GCPV method is, currently, the only genome comparison based method that allows for the characterization of relationships between gene clusters based profiles of individual genes in clusters.

**Results:**

The GCPV method groups together reasonably related operons in *E. coli* about 60% of the time. The method is not sensitive to the choice of a reference genome set used and it outperforms the conventional phylogenetic profiles method. Finally, we show that the method works well for predicted gene clusters from *C. crescentus* and can serve as an important tool not only for understanding gene function, but also for elucidating mechanisms of general biological processes.

**Conclusions:**

The GCPV method has shown to be an effective and robust approach to the prediction of functionally related gene sets from proximity-based gene clusters or operons.

## Background

### Introduction

Next generation sequencing technology has caused an explosion in the genomic data that is available to the research community. As a consequence, the annotation of such large numbers of diverse genomes has now become a major challenge. More specifically, in order to elucidate novel biological processes in certain organisms, assigning functions to the genes involved and understanding their interplay have become key problems. To address these challenges, several methods of function assignment have been proposed over the past decade. These range from simple homology-based strategies (BLAST [1], bi-directional best hits, etc.) to comparative genomics-based methods such as the gene cluster method [2], the phylogenetic profiles method [3], and the gene fusion [4] method.

In the case of prokaryotes, the gene cluster method has been very effective as functionally related genes tend to be physically clustered together on the genome and these arrangements tend to be conserved due to selection pressures [5]. However, conservation of genes is highly sensitive to the set of reference genomes and their phylogenetic relationship. In general, gene clusters are correct, in terms of functional relationship of component genes, but gene clusters are small and fragmented when clusters conserved in many genomes are sought. In addition, proximity-based clusters are often fragmented since not all genes in a functionally related gene set are physically co-located.

Another technique called the phylogenetic profiles method has been effective for co-evolving gene sets. However, this method is prone to false positives. Moreover, both these methods do not help understand the underlying mechanisms of biological processes. In order to establish such higher-level functions for both genes and gene clusters, we adopt a novel approach that involves the identification of functionally related gene clusters. To the best of our knowledge, this identification problem has not been addressed before. In this paper, we propose a novel method called the Gene Cluster Profile Vector (GCPV) method that combines strength of both techniques. GCPV takes, as an input, a set of gene clusters that are stringently defined and then group clusters based on the similarity of two clusters defined as the occurrence profiles of individual genes of the clusters in a set of genomes. We evaluate the GCPV method’s effectiveness in grouping together related operons in *Escherichia coli* and also assess the performance of the GCPV method in comparison to the single-gene phylogenetic profiles method. We then test its performance on predicted gene clusters from *Caulobacter crescentus*.

### Motivation

Genes whose products that contribute to the same biological process tend to form small clusters in prokaryotes. However, these clusters themselves, tend to be spread out through the entire genome. Simple proximity-based methods would be unable to identify functional relatedness in such cases. Moreover, although certain biological processes are observed in certain organisms, the corresponding clusters related to them may be fragmented into smaller clusters. On the other hand, although the phylogenetic profiles method has been successfully used for gene function assignment, it is based on the assumption that genes with related functions would have similar evolutionary profiles. This assumption tends to lead to false positives due to spurious matches. We propose a method that combines the advantages of the proximity and evolutionary constraints inherent in these two methods. Previous attempts at combining these methods, used individual scores for a gene, based on proximity and on co-evolution and combined these scores to assign it a function [6]. Since the goal of this study is not only to assign specific functions to genes but to also identify functional relationships between whole gene clusters, such score-based methods cannot be used. We, therefore, propose a more intuitive combination of the phylogenetic profiles and gene cluster methods.

### Problem description

The problem being addressed in this paper can be described as follows. Given a target genome, a set of gene clusters in the target genome (as predicted by any cluster prediction algorithm [7-11]) and a reference genome set, the goal is to identify functionally related gene clusters in the target genome and thus, generate clusters of gene clusters that contain gene clusters with similar biological functions, i.e.

**Input:** (1) *G_T_* , a target genome; (2) ***C***, a set of gene clusters predicted by a proximity-based method in *G_T_* ; (3) ***G***, a set of reference genomes that are at varying evolutionary distances from *G_T_*.

**Output: *L***, a set of clusters of gene clusters where each cluster contains functionally related gene clusters. The main reason behind the use of proximity-based gene clusters as input is that, gene clusters, when predicted with stringent parameters, are generally accurate but are fragmented as small sets. Thus, we have designed the GCPV method to group together such tight, fragmented clusters.

### Challenges

In order to address the above problem, several challenges have to be overcome. First, no prior information on gene or cluster functions is known. Second, in order to identify functionally related gene clusters, both proximity and conservation information in these clusters need to be considered. Third, when considering phylogenetic profiles of gene clusters, the method needs to be independent of the size of gene clusters. Finally, comparative genomics methods like the phylogenetic profiles methods are typically dependent on the size and nature of the reference genome set used and such dependence needs to be minimized. Thus, the design and implementation of a novel method to identify functionally related gene clusters is not trivial.

## Results and discussion

### Evaluating the GCPV method - operons from E. coli

The best approximation for a gene cluster is an operon as operons are also sets of genes that are constrained by proximity to each other. However, it must be noted that an operon corresponds to a set of co-transcribed and co-regulated genes. We have used a set of known operons in *Escherichia coli* K-12 substr. MG1655 (NCBI RefSeq: NC_000913) that have been verified by experiments. This dataset was obtained by filtering out computationally predicted operons from RegulonDB (Release 6.7) [12]. Subsequently, there were 1299 genes represented in 379 operons from *E. coli.* This data, along with single gene phylogenetic profile information was input into the GCPV workflow. Note that for this and all the following experiments, only prokaryotic species were used as reference genomes and these were downloaded from NCBI. The resulting clusters of operons were evaluated using SEED broad categories.

**Figure 1:**
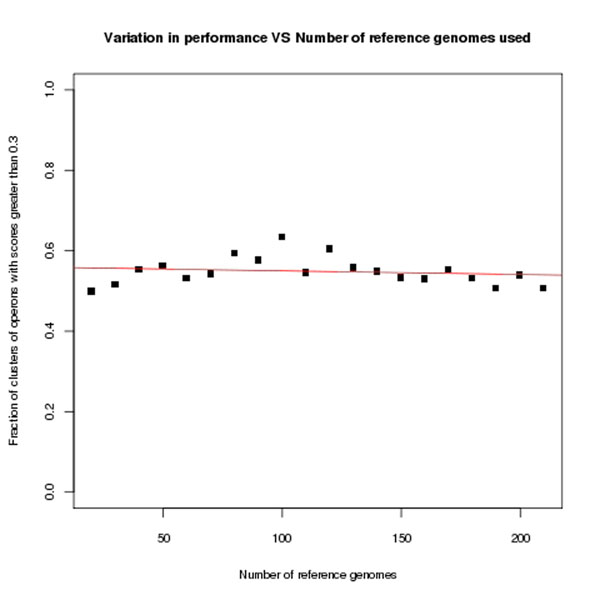
**Correlation of the validation scores of clusters of operons obtained by the GCPV method with the number of genomes used in the original reference set** The x-axis represents the reference genome set size and the y-axis represents the fraction of clusters of operons with scores greater than 0.3. The slope of the line in the figure is -0.00008997 which indicates that there is very little dependence between the two variables.

We found that, on average for each starting reference set (ranging from 20 to 210 genomes), about 87 clusters of operons were produced. About 60% of the clusters of operons had scores of at least 0.3 at the broad category level (Fig. 1) and about 40% at the SEED subsystem level (which is more specific as it comprises natural language identifiers). Note that we have used a less stringent cutoff simply because our evaluation method itself is harsh on smaller clusters (especially clusters containing merely three operons). Fig. 1 also shows that there is very little correlation between performance of the method and the number of genomes used in the original reference set. As can be seen in Additional File 1, clusters of operons are fairly consistent across reference sets of different sizes. Although some of these clusters show slight differences in their members, there tend to be a core set of operons common to all genome sets. There are also a number of clusters that are unique to each set. These tend to be smaller clusters broken down from larger clusters of operons. We believe both these issues can be explained by the fixed tree-cut height we use during hierarchical clustering.

**Figure 2:**
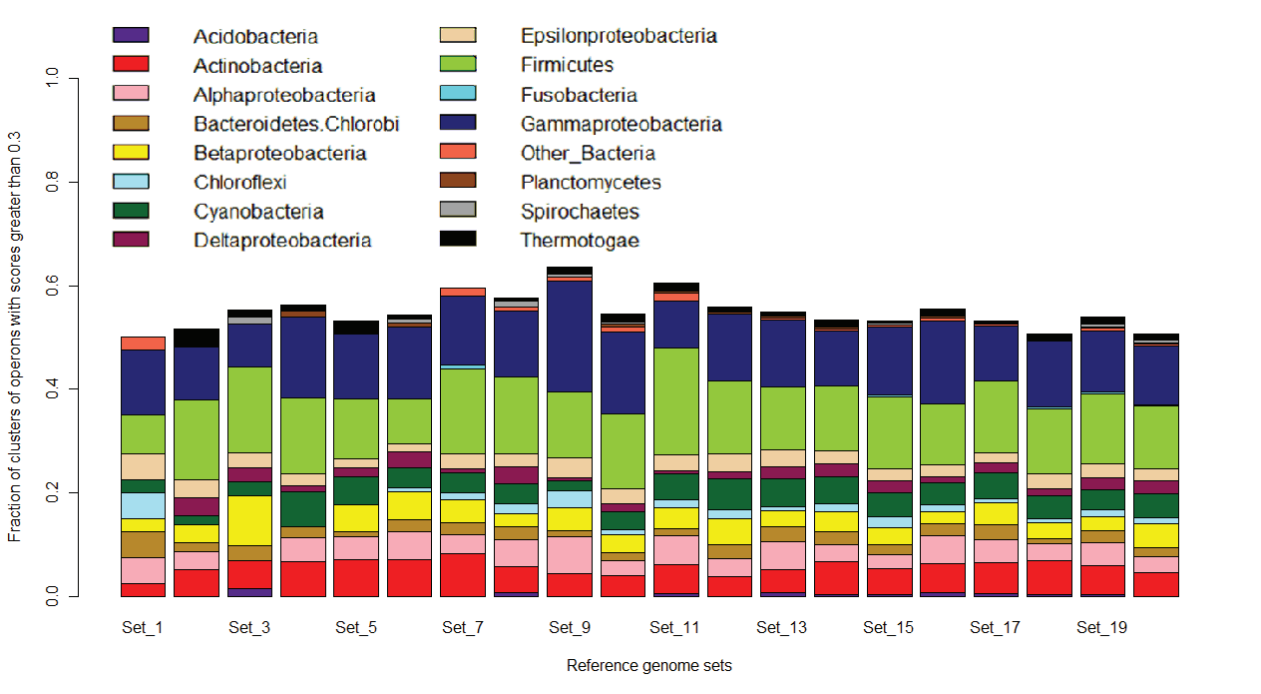
**Breakdown of the reference sets in Fig. 1 and their effect on the results from the GCPV method** The x-axis represents the 20 starting reference genome sets and the y-axis is the same as in Fig. 1. Although further studies are required, this plot does indicate that the nature of the reference set does not affect the GCPV method drastically.

In order to get some sense of the effect of the phylogenetic relationships among genomes in different reference sets, we broke the reference sets in Fig. 1 down to the phylogenetic classes of each genome. Fig. 2 shows that although in some reference sets, all classes are not represented, the results are still similar. Note that these original sets were randomly devised and certain classes like Gammaproteobacteria and Firmicutes are well-represented in all sets due to inherent biases in public databases.

### Comparing performance with single-gene phylogenetic profiles method

**Figure 3:**
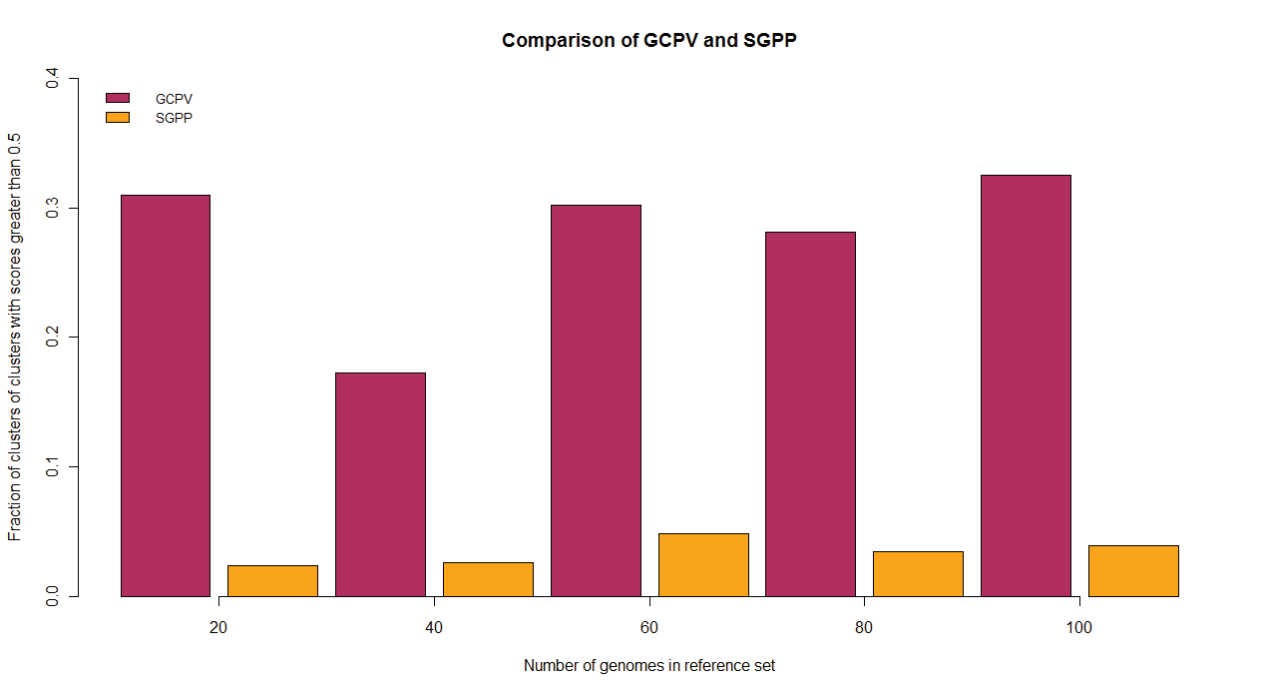
**Comparison of the GCPV (maroon) and the SGPP (orange) methods for a dataset of known operons and artificial gene clusters in E. coli** The x-axis represents the reference genome set size and the y-axis represents the fraction of clusters of clusters with scores greater than 0.5.

We further sought to test the usefulness of the GCPV method in the characterization of novel genes. For this, a comparison was made with respect to an in-house implementation of the single-gene phylogenetic profiles (SGPP) method. The same dataset and the same validation approach was used as in the benchmarking tests. However, in order to make a fair comparison, the dataset of operons was mixed with ‘artificial’ gene clusters (containing at least three genes) generated with intra-cluster distance cutoff of 300 bp [2]. This increased the number of genes in the dataset to 3709, which is closer to the total number of genes in the genome. Additionally, the evaluation method was slightly changed to enable validation at the gene level as opposed to the cluster level. This was achieved by skipping the step where the union of categories of genes within a cluster were assigned to the whole cluster (See Evaluation subsection in Methods). As shown in Fig. 3, on average, the GCPV method performs 10 times better than the SGPP method for identical reference genome sets even with a strict cutoff of 0.5. The SGPP implementation is as described in Step 1 in the Methods section and is a baseline implementation. Although subsequent work has improved upon the SGPP method through different variants of the method, we believe if similar variations were to be applied to the GCPV method, its performance would improve similarly.

### Testing the GCPV method - gene clusters from C. crescentus

**Figure 4:**
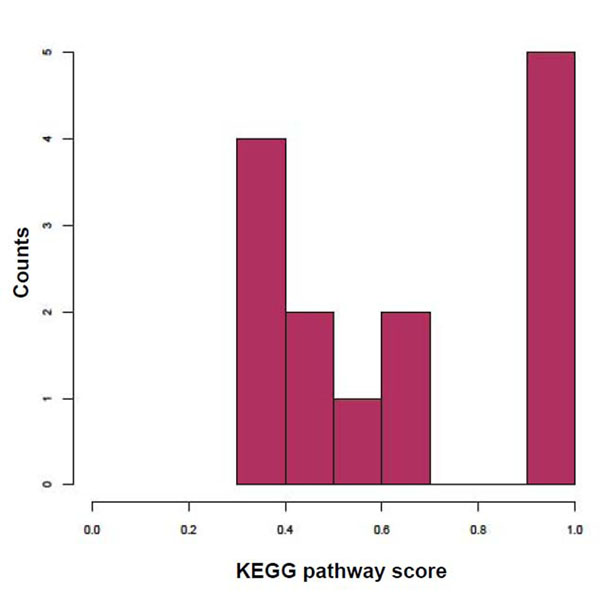
Distribution of KEGG pathway scores for clusters of gene clusters predicted by the GCPV method in C. crescentus

For this experiment, we used gene clusters from *Caulobacter crescentus* NA1000 (NCBI RefSeq: NC_011916), predicted to be conserved in four other members of the Alphaproteobacteria class by the PhyloEGGS algorithm (unpublished). This dataset contained 300 genes represented in 66 gene clusters. A reference genome set of size 100 was used and evaluation was done using KEGG pathway information [13]. This was done because the target genome was relatively new and no SEED information was available for it at the time of the study. Since this dataset was much smaller, a lower number of clusters of gene clusters was obtained (14 clusters). Of these 14 predicted clusters, about 79% of them showed scores greater than 0.5 (Fig. 4). This indicates that the GCPV method does group together functionally related gene clusters effectively and can be used on larger datasets with gene clusters that are not well-characterized.

### Case studies

An interesting example is the CCNA_02239 gene in *C. crescentus* which is annotated as a hypothetical protein. PhyloEGGS predicted it to be a part of a two-gene cluster also containing a translation initiation inhibitor (CCNA_02241). The GCPV method included this gene cluster in a cluster of cluster of size three with the remaining two clusters being assigned similar KEGG pathways (ccs00190 and ccs01100). The latter is a general metabolic pathway while the former deals with oxidative phosphorylation. Based on this, one can conclude that CCNA_02239 may play a role in oxidative phosphorylation or a similar pathway. This would not have been evident through a simple BLAST search or by just using the cluster context. This highlights the use of the GCPV method as an effective annotation tool.

Another case study involves genes responsible for the various steps in the flagellar assembly process. Table 1 shows the different clusters of operons that contain flagellum-related operons from *E. coli.* Cluster 23 seems to be the most specific to flagellar assembly while the other clusters do not entirely contain operons involved in flagellar assembly. Interestingly, the justification for the fact that *flgAMN*, *flhDC* and *fliDST* are not grouped into cluster 23 along with the other operons is that these operons consist of genes that produce early or late gene products in the flagellar assembly process. Thus, the GCPV method also enables the possible distinction between genes that contribute to a process at different time stages. The *fliAZY* operon is isolated probably because it has been established that the *fliZ* and *fliY* genes from this operon are not required for motility [14]. Thus, the GCPV method also enables the identification of genes that are involved but are perhaps not instrumental to a particular process.

**Table 1:** Clusters of operons from E. coli with a reference genome set of 120 genomes

Identifier for cluster of operon	Operons
Cluster 13	*ackA-pta*, *argT-hisJQMP*, *artPIQM*, ***fliAZY***, *glnHPQ*, *metNIQ*

Cluster 40	*fimAICDFGH*, ***flgAMN***, ***flhDC***, *slp-dctR*, *smtA-mukFEB*

Cluster 43	***flgBCDEFGHIJ*****,** ***flgKL*****,** ***flhBAE*****,** ***fliLMNOPQR*****,** ***motAB-cheAW***

Cluster 163	*csgDEFG*, ***fliDST***, *yeaGH*

Operons involved in flagellar assembly are marked in bold and identifiers are those in Additional File 1

## Conclusions

We have established an effective and robust method for the detection of functionally related gene clusters and thus, genes, with no prior information on function provided. The GCPV method shows minimum dependence on the reference genome set used and has been shown to outperform the basic phylogenetic profiles method which is in agreement with previous work in this area [15]. However, our work serves as an improvement over the work in [15] as our method can accommodate gene clusters of any size. A limitation of the GCPV method is that genomic coverage may not span the entire genome as not all genes are present in as parts of clusters.

This was aimed to be a pilot study and future work includes the use of more sophisticated clustering techniques to further improve performance. This is particularly important in the context of improving consistency at the individual cluster level. Interestingly, in its current form, the GCPV method still groups together functionally similar clusters/operons as indicated by the evaluation scores. This implies that although individual clusters may not be reference-set independent, the GCPV method generates clusters containing functionally related gene clusters and can be used for function assignment. Additionally, the GCPV method can be adapted to any gene set other than proximity-based gene clusters, as long as the intra-set coupling is tight. In general, the GCPV method holds the potential to play a role not only in genome annotation but also in testing hypotheses for roles of previously uncharacterized genes in metabolic pathways, protein-protein interactions and general biological processes.

## Methods

### Description of the Gene Cluster Profile Vector method

**Figure 5:**
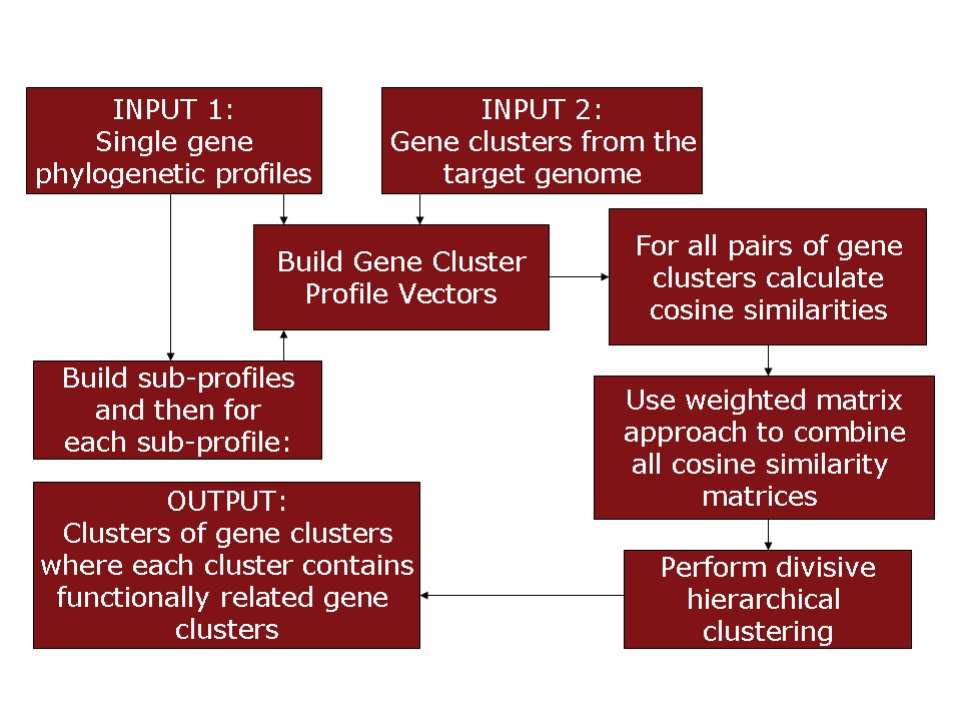
Schematic representation of the Gene Cluster Profile Vector method

The basic idea behind the GCPV method involves the use of phylogenetic profiles of whole gene clusters. These vectors serve as numerical representations of both proximity and conservation information. Each step of the GCPV workflow is discussed in detail below and the workflow itself is illustrated in Fig. 5.

#### Step 1: Construction of Gene Cluster Profile Vectors

**Figure 6:**
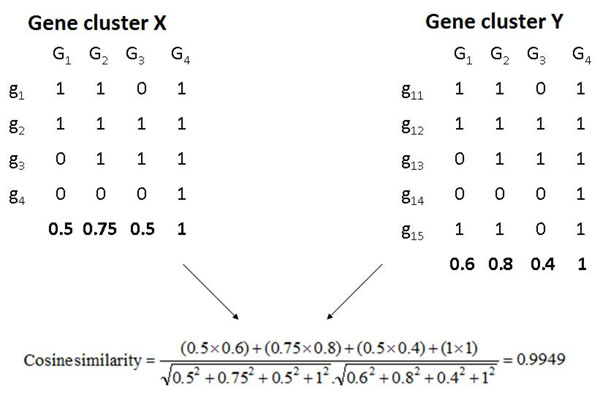
**Schematic representation that depicts the construction of profile vectors for two hypothetical gene clusters and the calculation of cosine similarity between them** Individual genes in each cluster are represented by *g* and the reference genomes used are represented by *G*. The vectors marked in bold are the resulting GCPVs.

In order to construct GCPVs, for a target genome, single gene phylogenetic profiles for all genes and gene cluster information need to be provided. For this study, a gene in the target genome is considered to be present in a reference genome if its single best BLAST match from that genome has an E-value lower than 1*E*^–10^[16]. Once the single gene phylogenetic profiles are built, they are grouped together based on the gene cluster information provided. Then, for each cluster, a GCPV is constructed based on the frequencies of the gene presences in the reference genomes. Formally, if there are *n* genes in a cluster, then:

where *a_j_* is the *j^th^* value in the GCPV and *b_ij_* is one if the gene is present in the *j^th^* genome & zero otherwise. Two examples of this are shown in Fig. 6.

Therefore, for a given gene cluster, its profile vector is a numeric vector where each element represents the extent of conservation of genes from that cluster in a specific reference genome. For example in Fig. 6 the third element in the GCPV on the left is 0.5, it means that the third genome contains only half the genes from that cluster.

#### Step 2: Comparison of GCPVs by cosine similarity calculations

For a given gene cluster, its profile vector provides a succinct numeric representation that allows for its comparison with profile vectors of other gene clusters in the target genome, i.e. GCPVs can be used in the calculation of a measure of functional relatedness or similarity between gene clusters. In the case of GCPVs, vectors with similar elements occurring in the same specific order would need to be regarded as being more related to each other than those with elements that break order. The cosine similarity measure is ideal for this condition to be satisfied. Moreover, cosine similarities have been used previously in the context of single gene phylogenetic profiles [6]. For two vectors  and  of sizes *m*, the cosine similarity is given by the following equation:

An example of this has been explained in Fig. 6 where two hypothetical gene clusters, one with four genes and one with five are considered. The cosine similarity of their GCPVs turns out to be 0.9949 which indicates a high similarity. This is reasonable because gene cluster *Y* is exactly identical to cluster *X* in its structure except for the addition of a fifth gene *g*_15_*.* It can also be observed that the profiles of *g*_11_ and *g*_15_ are identical to each other. Thus, this fifth gene does not affect the conservation profile of the cluster as a whole and this results in a high cosine similarity value.

#### Step 3: Generation of sub-profiles

As mentioned earlier, one of the key challenges in a comparative genomics method is reducing the dependence of the method on the reference genome set used. In order to address this challenge, an intermediate step has been incorporated into the GCPV workflow. This step basically involves the random generation of new reference genome subsets from the original reference genome set. These subsets vary in size (with the original reference genome set being the largest) and result in the generation of GCPVs of different lengths. For a given reference genome set, these can loosely be termed as ‘sub-profiles’ and cosine similarities can be calculated for GCPVs generated from each of these sub-profiles. In these study, the minimum sub-profile size was 12 and the increase in profile size occurred in increments of 12.

The idea behind this is that during the subsequent steps in the workflow, these cosine similarities would be combined to improve the method in two ways. First, as mentioned earlier, the use of cosine similarities from reference genome subsets would help reduce the dependence of performance on the reference genome set. Second, by randomly generating smaller reference genome subsets, one can account for lineage-specific co-evolution whose effects often arise in the phylogenetic profiles method.

#### Step 4: Construction of cosine similarity matrices and the weighted matrix approach

The steps outlined previously result in a single value between zero and one for a given pair of gene clusters within the target genome. This is done for all pairs of gene clusters to generate a cosine similarity matrix. If there are *k* gene clusters, this matrix would have *k* rows and *k* columns with all entries in the diagonal being unity (since the cosine similarity calculated for a gene cluster and itself is one). This is repeated for each reference genome subset and would result in many such cosine similarity matrices.

In order to meaningfully combine these matrices to provide clearer functional relationships between gene clusters, a weighted average approach is adopted. Each cosine similarity matrix is weighted based on the phylogenetic relationships between each of the reference genomes used and the target genome. More specifically, a weight for a matrix is the mean of the normalized phylogenetic distances (16S rRNA sequence dissimilarities) between each reference genome and the target genome. This calculation is done by the APE package [17]. This method of weight assignment is most intuitive when considering the fact that one of the aims is to reduce the effects of phylogeny on the results of the GCPV method. Mathematically, the entire procedure can be written as:

where *M* is the final combined cosine similarity matrix, *M*^(^*^i^*^)^ is the cosine similarity matrix resulting from the reference genome subset *i* and *w_i_* is the weight for the matrix *M*^(^*^i^*^)^.

#### Step 5: Divisive hierarchical clustering

The final result of the GCPV method is a reasonable grouping of gene clusters that reflects their functional relatedness to each other. After having experimented with various clustering techniques, it was found that density-based methods fail because cosine similarities seem to be distributed evenly and the Markov Cluster method results in a smaller number of larger clusters. The divisive hierarchical clustering technique seems to give the most reasonable results. It basically assumes that all data points belong to one large cluster and then proceeds to break the cluster down into smaller units based on intra-cluster and inter-cluster distances.

### Evaluation method

In order to establish the effectiveness of the GCPV method, we have performed experiments on both real and predicted data. For validation purposes we have used either the SEED [18] or the KEGG [13] broad categories. The validation methodology involves the calculation of a score for each cluster of cluster generated.

Mathematically, the validation procedure can be explained as follows. Consider a cluster of gene clusters *C* containing *K* gene clusters denoted by *c*_1_, *c*_2_,…, *c_K_*. Let the number of genes in each cluster be *n*_1_, *n*_2_,…, *n_K_*. Let the broad categories for one such gene cluster *c_x_* by denoted by *B_c_x__* and for its genes be denoted by *B*_*g*_1__, *B*_*g*_2__,…, *B_g_n__x__.* Then:

Now, once all *K* gene clusters have been assigned a set of broad categories, the score for cluster of gene cluster *C* is calculated as follows:

## Authors contributions

SK devised and developed the method and also designed the experiments. VRP devised and developed the method and performed the experiments. Both authors prepared the manuscript.

## Competing interests

The authors declare that they have no competing interests.

## Supplementary Material

Additional file 1 **Comparison of clusters of operons generated for E. coli** Each entry corresponds to a cluster of operon for the particular reference set size. Clusters from each set were manually compared and placed together in the same row. The clusters are mostly reproduced even with changes in set size. Most of the clusters unique to certain reference sets are a result of cluster-splitting due to the fixed tree-cut height used during hierarchical clustering. Click here for file
